# The Role of Endocarditis, Myocarditis and Pericarditis in Qualitative and Quantitative Data Analysis

**DOI:** 10.3390/ijerph6122919

**Published:** 2009-11-26

**Authors:** Norman Schöffel, Karin Vitzthum, Stefanie Mache, David A. Groneberg, David Quarcoo

**Affiliations:** Institute of Occupational Medicine, Charité-Universitätsmedizin Berlin, Free University Berlin and Humboldt-University Berlin, Thielallee 69- 73, D-14195 Berlin, Germany; E-Mails: norman.schoeffel@charite.de (N.S.); stefanie.mache@charite.de (S.M.); david.groneberg@charite.de (D.A.G); david.quarcoo@charite.de (D.Q.)

**Keywords:** endocarditis, myocarditis, pericarditis, scientometrics, h-index, self-citation, impact factor, coauthor ship

## Abstract

The current study is the first scientometric analysis of research activity and output in the field of inflammatory disorders of the heart (endo-, myo- and pericarditis). Scientometric methods are used to compare scientific performance on national and on international scale to identify single areas of research interest. Interest and research productivity in inflammatory diseases of the heart have increased since 1990. The majority of publications about inflammatory heart disorders were published in Western Europe and North America. The United States of America had a leading position in terms of research productivity and quality; half of the most productive authors in this study came from American institutions. The analysis of international cooperation revealed research activity in countries that are less established in the field of inflammatory heart disorder research, such as Brazil, Saudi Arabia and Tunisia. These results indicate that future research of heart inflammation may no longer be influenced predominantly by a small number of countries. Furthermore, this study revealed weaknesses in currently established scientometric parameters (*i.e.*, h-index, impact factor) that limit their suitability as measures of research quality. In this respect, self-citations should be generally excluded from calculations of h-index and impact factor.

## 1. Introduction

Inflammation of the heart can be classified as endocarditis, myocarditis or pericarditis, depending on the tissue affected. These disorders can lead to sudden cardiac death and/or chronic heart insufficiency [1–3]. They are associated with a high mortality rate (e.g., cardiac valve insufficiency after endocarditis). The numbers of publications about endocarditis, myocarditis and pericarditis have grown considerably, especially in the past 20 years. The resulting informational overload makes it difficult for researchers and cardiologists to read and interpret all relevant publications of scientific importance.

Scientometrics is a relatively new discipline that measures scientific output and can help medical professionals to evaluate the distribution and quality of research accomplishments in a given field [4,5]. Scientometrics enables us to measure and analyze scientific publications in terms of quality and quantity. Nevertheless, to date there has been no scientometric evaluation of endocarditis, myocarditis and pericarditis research. Therefore, the objective of the present study was to determine and to compare research efforts in each of these topics in terms of the following characteristics: annual number of published items, their country of origin, items’ citation rate per country and the most productive and prolific journals and authors. These characteristics were determined by large-scale data analysis, scientometric approaches and density-equalizing mapping.

## 2. Experimental Section

### 2.1. Data Collection

Data were retrieved from the ISI Web of Science database as described in a recent publication [6,7].

### 2.2. Search Strategies

Each of the terms “endocarditis”, “myocarditis” and “pericarditis” were entered in the search field respectively and combined with Boolean operators (*i.e.*, AND and OR) to assess the overall number of published items by using the “analyze” and the “citation report” functions.

### 2.3. Time Span

All items published between 1900 and 2007 were included in this analysis. Results from 2008 and 2009 were excluded due to incomplete database indexing at the time of assessment.

### 2.4. Citation Quantities

The items published about each topic were analyzed by the “citation report” method. Results show the average number of citations each publication received in a country-specific manner. The average citations-per-item analysis may lead to distorted results, as the average citation rates of countries with relatively few items appear disproportionately high. Therefore, a threshold of 30 published items was chosen to provide representative results.

### 2.5. Data Categorization

All data files were analyzed according to the following aspects: their country of origin, cooperation among countries, the most productive journals, the date of publication and the most productive authors. Data were automatically transformed into Excel files and visualized as diagrams.

Global geographical and political changes over the study period, which could pose systematic limitations, were accounted for in our study. Thus, publications with the following origins were reclassified as United Kingdom: England, Northern Ireland, Wales and Scotland, (UK). Publications from “WEST GERMANY”, “FED REP GER”, “GER DEM REP” and “BUNDES REPUBLIK” were reclassified as “Germany”. Publications from the former countries Yugoslavia, Czechoslovakia and USSR were traced to their institution of origin and reassigned to the current country of the institution’s location.

### 2.6. Density-Equalizing Mapping

The distribution of the total number of published items and average citation rates per country were visualized by applied calculations of Gastner and Newman’s algorithms. Thereby territories were resized according to a particular variable, *i.e.*, the number of published items and the average citation rate according to a recently published method [8]. The area of each country was scaled in proportion to its total number of published items regarding one or more of the inflammatory heart disorders: endocarditis, myocarditis and pericarditis. The same method was applied to illustrate the average citations rate of each country [8].

### 2.7. H-Index

The h-index is a measure of researchers’ scientific quality. Scientists’ h-indices depend on how many items they have published and on how often those articles have been cited. For instance, an author who published ten articles, each of which was cited at least ten times, has an h-index of ten. If only seven of the ten have been cited at least seven times each, the h-index would be seven. If an author has only published two articles, which have been cited more than ten times each, the h-index would still be two [9].

### 2.8. Impact Factor

A journal’s impact factor is defined as the number of citations per year in relation to the number of articles published in the two preceding years [10]. The impact factor was used to compare the most productive journals.

### 2.9. Analysis of International Cooperation

To visualize the international cooperation, data of references of all publications referring to each of the search terms (*i.e.*, “endocarditis”, “myocarditis” and “pericarditis”) were stored as plain text files and analyzed. Cooperation was defined as the collaboration of two or more authors from different countries for a single publication.

## 3. Results and Discussion

### 3.1. Total Number of Published Items

**Figure 1.**
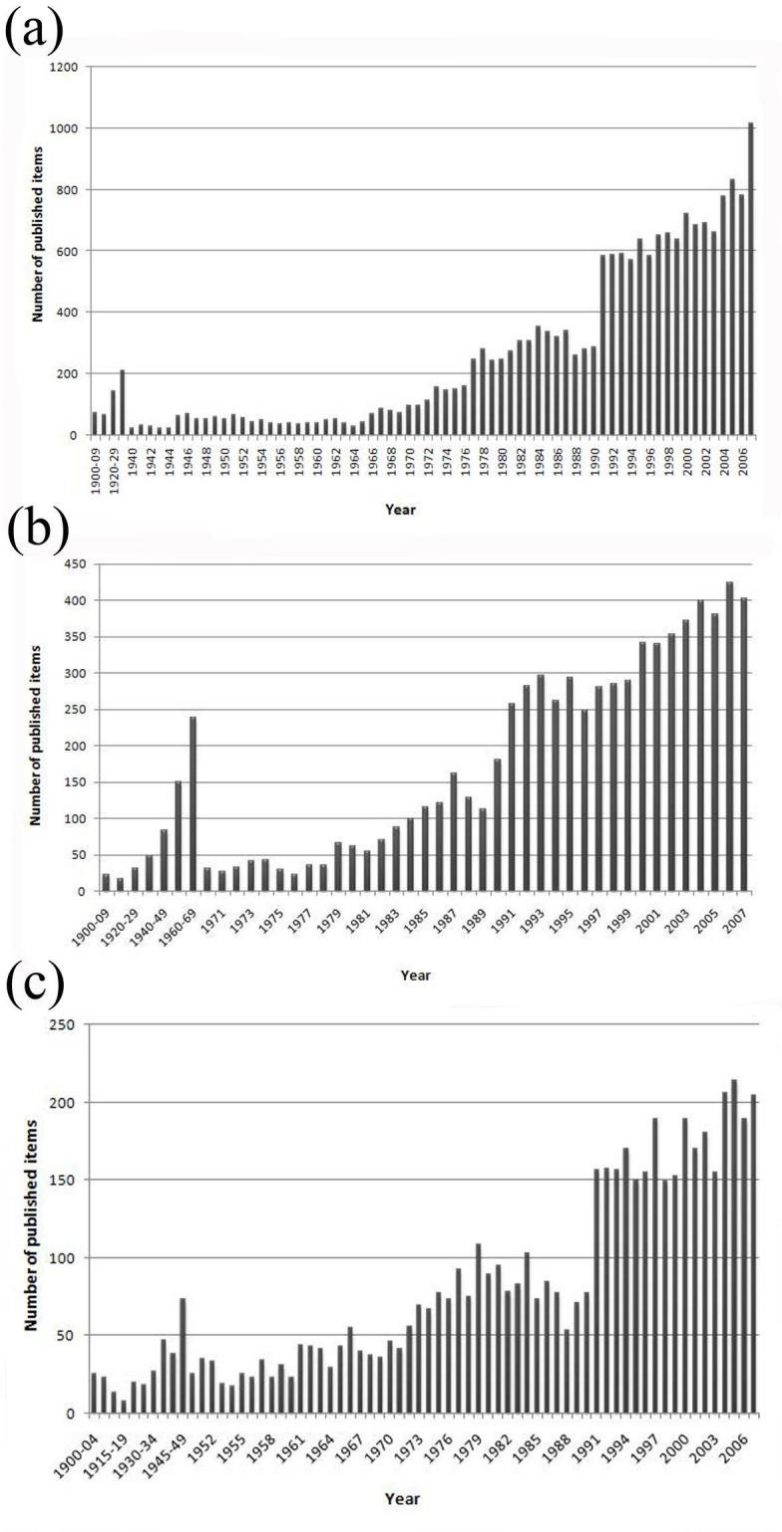
(a) Publication numbers for endocarditis. (b) Publication numbers for myocarditis. (c) Publication numbers for pericarditis.

From 1900 to 2007 over thirty-two thousand publications pertaining to the three inflammatory disorders of the heart were included in the Web of Science database. A total of 18,967 publications were found for “endocarditis” (Figure 1a), 7,803 publications for “myocarditis” (Figure 1b) and 5,552 publications for “pericarditis” (Figure 1c).

### 3.2. Analysis of Origin and Cooperation

**Figure 2.**
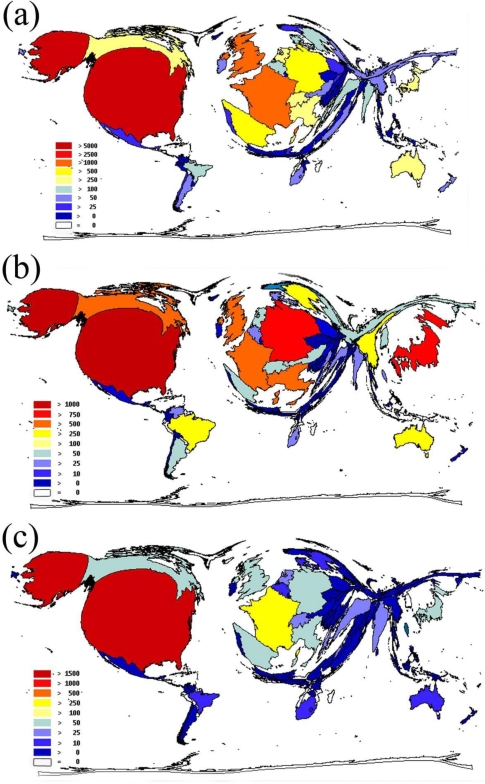
(a) Analysis of origin and cooperation: Density equalizing mapping for endocarditis. (b) Analysis of origin and cooperation: Density equalizing mapping for myocarditis. (c) Analysis of origin and cooperation: Density equalizing mapping for pericarditis.

The 18,967 entries for “endocarditis” originated from 107 countries—43.9% of these items were published in the USA, France and the UK (Figure 2a). The 7,803 entries dealing with myocarditis were published in 97 countries. The USA, UK and Canada were the most productive countries, representing 35.2% of all items in the set (Figure 2b). The 5,552 publications about pericarditis originated from 86 countries. The USA, France and the UK were again the most productive ones, accounting for 37.2% of all published items (Figure 2c). The dominance of the United States in inflammatory heart disorder research is illustrated by density-equalizing mapping (Figure 2a, 2b and 2c).

### 3.3. Cooperation Analysis

**Figure 3.**
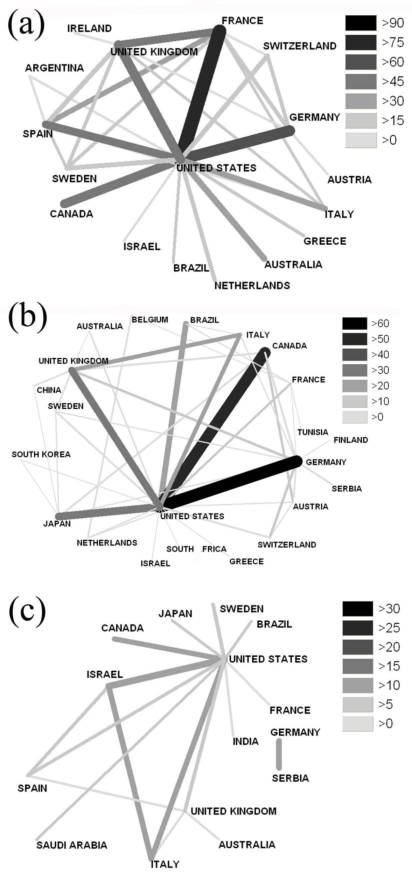
(a) Cooperation analysis regarding the term “endocarditis”. (b) Cooperation analysis regarding the term “myocarditis”. (c) Cooperation analysis regarding the term pericarditis.

The analysis of international cooperation yielded the following results:

Collaborative efforts between the USA–France (85), USA–Germany (71), USA–UK (58) and USA–Canada (56) were the most productive for endocarditis research (Figure 3a). Regarding the collaboration for publications dealing with myocarditis, the cooperation between the USA–Germany (62), USA–Canada (59), USA–UK (36) and USA–Japan (41) can be considered the most productive (Figure 3b).

Cooperation analysis for pericarditis revealed the cooperation between the USA–Israel (14), Canada–USA (11), Serbia–Germany (12), USA–Italy (12) as the most productive (Figure 3c).

Furthermore, some countries, e.g., Tunisia, Brazil, and Saudi Arabia, had detectable results in the analysis of international cooperation although they are not as well-established in the scientific field (Figure 3a, 3b and 3c).

### 3.4. Citation Parameters

**Figure 4.**
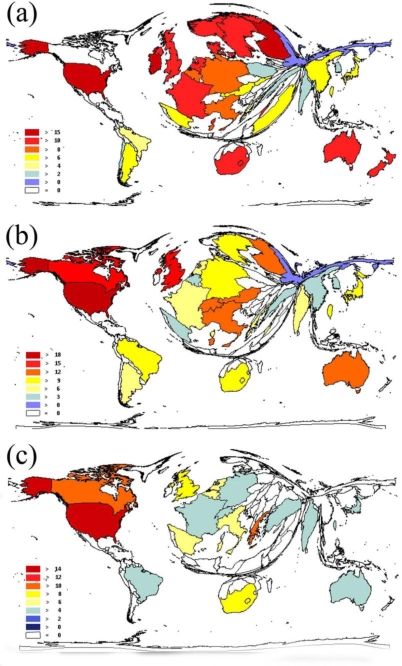
(a) Citation parameters visualized through density equalizing techniques for endocarditis. (b) Citation parameters visualized through density equalizing techniques for myocarditis. (c) Citation parameters visualized through density equalizing techniques for pericarditis.

The United States had the highest average citation rate of 19.10 in the country-specific analysis of endocarditis research, followed by Finland (17.06) and Ireland (15.29) (Figure 4a).

The USA (18.84), the UK (18.15) and Canada (17.34) showed the highest average citations rates for myocarditis publications (Figure 4b). Regarding pericarditis research, the USA (14.16) was found to have the highest rate, followed by Canada (11.08) and Greece (11.02) (Figure 4c).

### 3.5. Analysis of the Most Productive Authors

**Figure 5.**
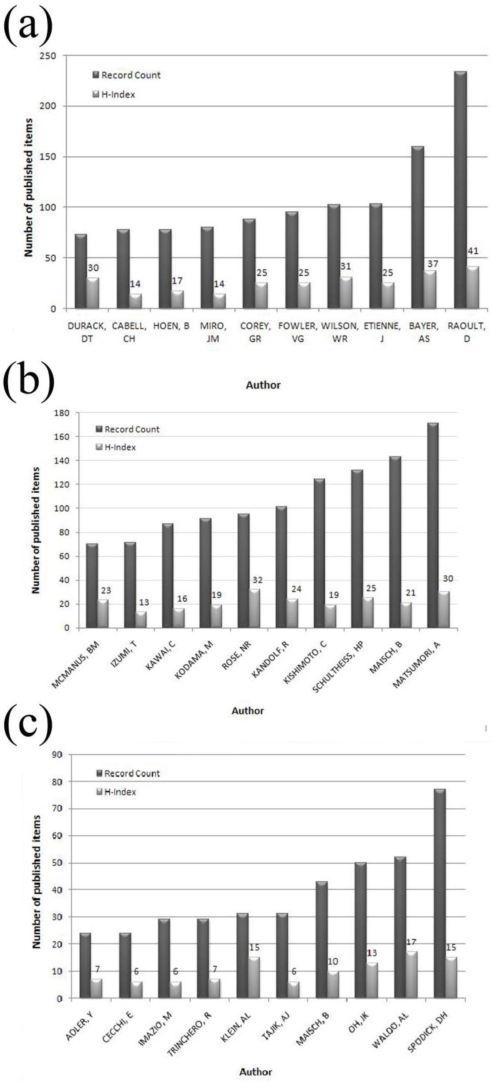
**(a)** Analysis of the most productive authors publishing “endocarditis” related articles. **(b)** Analysis of the most productive authors publishing “myocarditis” related articles. **(c)** Analysis of the most productive authors publishing “pericarditis” related articles.

Individual research output was analyzed by authors’ total number published items and respective h-indices. The ten most productive authors of were identified for each inflammatory heart disorder (Figure 5). Regarding endocarditis research, “RAOULT, D” was the most productive author with 234 items, followed by “BAYER, AS” with 160 items and “ETIENNE, J” with 103 items. However, the h-index analysis showed a different distribution—“RAOULT, D” had the highest h-index (41), followed by “BAYER, AS” (37) and “WILSON, WR” (31) (Figure 5a).

“MATSUMORI, A” was the most productive author in the myocarditis analysis with 171 publications, followed by “MAISCH, B” (143 publications) and “SCHULTHEISS, HP” (132 publications). The ranking of h-indices showed a different distribution. “ROSE, NR” held the highest h-index (32), followed by “MATSUMORI, A” (30) and “SCHULTHEISS, HP” (25) (Figure 5b).

“SPODICK, DH” was the most productive author (77 publications) of pericarditis publications, followed by “WALDO, AL” (52 publications) and “OH, JK” (50 publications). Again the h-index showed a different distribution of research output. “WALDO, AL” had the highest h-index (17), followed by “KLEIN, AL” (15) and “SPODICK, DH” (15) (Figure 5c).

### 3.6. Analysis of Journal Impact Factors

**Figure 6.**
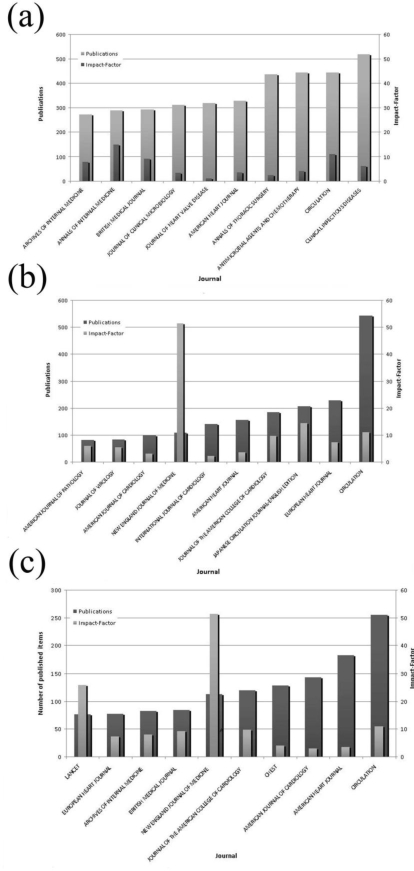
(a) Analysis of journal impact factors for the subject area “endocarditis”. (b) Analysis of journal impact factors for the subject area “myocarditis”. (c) Analysis of journal impact factors for the subject area “pericarditis”.

The most prolific journals for endocarditis research were “CLINICAL INFECTIOUS DISEASES” with 517 publications and an impact factor of 6, followed by the journals “CIRCULATION” (443 publications, impact factor 11) and “ANTIMICROBIAL AGENTS AND CHEMOTHERAPY” (443 publications, impact factor 4) (Figure 6a).Journal analysis for “myocarditis” showed “CIRCULATION” to be the most prolific source (542 publications, impact factor 11), followed by the journals “EUROPEAN HEART JOURNAL” (229 publications, impact factor 7) and the “JAPANESE CIRCULATION JOURNAL-ENGLISH EDITION” (207 publications, impact factor 2) (Figure 6b).

Regarding pericarditis research, “CIRCULATION” lead the ranking with 255 publications and an impact factor of 11, followed by the journals “AMERICAN HEART JOURNAL” (183 publications impact factor 4) and the “AMERICAN JOURNAL OF RADIOLOGY” (143 publications, impact factor 3) (Figure 6c).

### 3.7. Discussion

The present study was designed to evaluate the quality and quantity of the scientific research for main types of inflammatory heart disorders (endo-, myo- and pericarditis) using scientometric approaches, density-equalizing mapping and large-scale data analysis. Data analysis in a country-specific manner showed that the USA maintains a leading position in this field of research. Between 35.2–43.9% of all published items were authored by researchers working in the USA. Most of the international cooperation included USA-based workgroups. The most productive institutions were located in the USA, which had the highest citation rate of all publishing countries. The dominant role of American research found in this study has been described in studies of research output in other scientific areas [6,7]. Interestingly, some countries, such as Brazil, Saudi Arabia and Tunisia, had detectable results in the analysis of international cooperation although they are not as well-established in the field. These results suggest that future research in the field of inflammatory heart disorders may no longer be dictated by a relatively small number of countries.

The growing interest in the field of inflammatory heart disorders is reflected by the rising number of scientific publications over the past century. A sharp increase in publishing has been seen since 1990—nearly two-thirds of all items found were published in the past two decades. These results indicate increased interest in endo-, myo-, and pericarditis research but do not necessarily correlate with advances made in these fields since 1990. Modified evaluation criteria for academic personnel in terms of career advancement and/or fundraising policy may lead to the urgent need to publish. A general increase in publication numbers can be detected in most subject areas [6,7]. The tendency of coauthor ship and the general availability of the Internet may account, in part, for the observed rise in scientific publishing [11,12]. Another trend is to publish original research or case reports not as a single scientist but as a study group; some scientists tend to publish small slices of their work such as multicenter trials over time or publish their work as editorial authors. These trends may indicate a weakening of the ethical code within the world of science in order to reach economic goals [13,14].

Furthermore, looking at the most productive authors (number of published items) and their scientific impact (h-index) in the field of endocarditis research, the French physician “RAOULT, D” had published most articles and had the highest h-index. According to the number of publications, five of the ten most productive authors dealing with items concerning endocarditis are not of US-origin. Considering the smaller number of research institutions in France, it is challenging to explain the reasons for the French prominence. However, the relatively small number of French institutions in comparison to American institutions may lead to a concentration of research efforts that might benefit the leader of the institution. Furthermore, in France prolific authors often lead institutions or research groups and, therefore, participate in the research work of several scientists [15,16].

Different bacterial, viral or fungal agents play an important role in the pathophysiology of endocarditis [17]. Since the early discoveries of Louis Pasteur, French scientists have been renowned for making important contributions to microbiological research [18]. The legacy of their work is evidenced by the high proportion of French publications in the research of inflammatory heart disorders (e.g., pericarditis: France has the second highest amount of publications).

Subsequently, we tested the hypotheses that an increased prevalence of endocarditis or pericariditis in the French public might spur a rising interest in this field of research. However, epidemiologic distribution analysis did not show a concentration of cases in France but revealed similar rates of inflammatory heart disorders for most developed countries. Therefore, increased prevalence of these disorders can not account for the French prominence in this field of research [19].

Comparing the number of published items, international cooperation, the average citation rate, some countries had disproportionately high results. For instance, Finland, Norway, and Denmark had unexpectedly high citations rates in the endocarditis analysis. Further analysis revealed a tendency toward high self-citation rates in these countries, which was also found for the USA and the UK Self-citation leads to above average citation rates and to the distortion of subsequent qualitative research assessment [6,9,10], which limits the merit of established qualitative parameters, such as the impact factor and the h-index. The total number of published items (measure of productivity) and the h-index (measure of quality) were calculated to evaluate the most important authors of inflammatory heart disorder publications. However, these results should also be regarded skeptically due to the increasing tendency of coauthor ship and self-citations among authors over the past few decades. Self-citations and coauthor ship are factored into the h-index even though these factors may favorably influence the assessment of scientists’ research output. Journal editors and reviewers could use their influence on publishing processes to counteract the growing trends of self-citation and coauthoring. Interpreting our results, it is apparent that the h-index and the journal's impact factor are not impartial, independent indicators of the quality of research output but rather vulnerable to misrepresentation by self-citation and coauthoring practices.

The ISI-Web database was selected for this analysis in order to gain the complete reference data that formed the basis of our scientometric analysis of inflammatory disorders of the heart. Although PubMed includes similar numbers of publications, the corresponding reference data are incomplete, precluding the comparison of findings between the two databases.

## 4. Conclusions

Although there have been no quantum leap in the diagnosis or treatment of endo-, myo- and pericarditis [20–22]), we expect that the numbers of heart inflammation publications will continue to rise in the future because modern scientific funding policies put pressure on scientists to publish more items than scientific progress warrants [4,9,14]. The resultant informational overload deems it necessary for researchers and clinicians to sift through the numerous publications and assess their merit. Scientometrics can be a useful tool for prioritizing relevant research, although more reliable qualitative factors, for instance, those that exclude self-citation and “mass coauthoring” should be investigated.
